# Bis(tetra­methyl­ammonium) oxalate monohydrate

**DOI:** 10.1107/S1600536809039099

**Published:** 2009-10-03

**Authors:** Yun-Xia Yang, Qi Li, Seik Weng Ng

**Affiliations:** a College of Chemistry, Beijing Normal University, Beijing 100875, People’s Republic of China; b Department of Chemistry, University of Malaya, 50603 Kuala Lumpur, Malaysia

## Abstract

In the crystal structure of the title hydrated salt, 2C_4_H_12_N^+^·C_2_O_4_^2−^·H_2_O, the two independent cations, the anion and the water mol­ecule all lie on special positions of *m* site symmetry. In both cations, the mirror plane passes through the nitrogen atom and two methyl groups; in the anion, the mirror plane passes through two carbon and two oxygen atoms. The anions and water mol­ecules inter­act by O—H⋯O hydrogen bonding, forming a chain running along the *b* axis.

## Related literature

For the crystal structure of tetra­methyl­ammonium hydrogen oxalate, see: Mascal *et al.* (2000 ▶).
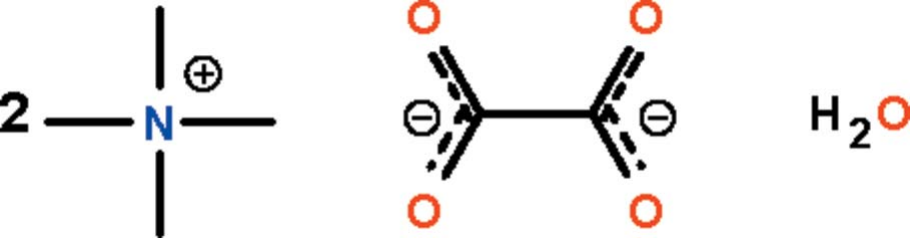


## Experimental

### 

#### Crystal data

2C_4_H_12_N^+^·C_2_O_4_^2−^·H_2_O
*M* *_r_* = 254.33
Orthorhombic, 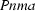

*a* = 24.614 (4) Å
*b* = 6.738 (1) Å
*c* = 8.633 (2) Å
*V* = 1431.8 (4) Å^3^
*Z* = 4
Mo *K*α radiation
μ = 0.09 mm^−1^
*T* = 293 K
0.50 × 0.10 × 0.10 mm

#### Data collection

Bruker APEX2 diffractometer
Absorption correction: none
3915 measured reflections
1367 independent reflections
1043 reflections with *I* > 2σ(*I*)
*R*_int_ = 0.024

#### Refinement

*R*[*F* ^2^ > 2σ(*F* ^2^)] = 0.053
*wR*(*F* ^2^) = 0.164
*S* = 1.01
1367 reflections
99 parameters
H atoms treated by a mixture of independent and constrained refinement
Δρ_max_ = 0.25 e Å^−3^
Δρ_min_ = −0.25 e Å^−3^

### 

Data collection: *APEX2* (Bruker, 2007 ▶); cell refinement: *SAINT* (Bruker, 2007 ▶); data reduction: *SAINT*; program(s) used to solve structure: *SHELXS97* (Sheldrick, 2008 ▶); program(s) used to refine structure: *SHELXL97* (Sheldrick, 2008 ▶); molecular graphics: *X-SEED* (Barbour, 2001 ▶); software used to prepare material for publication: *publCIF* (Westrip, 2009 ▶).

## Supplementary Material

Crystal structure: contains datablocks global, I. DOI: 10.1107/S1600536809039099/xu2618sup1.cif

Structure factors: contains datablocks I. DOI: 10.1107/S1600536809039099/xu2618Isup2.hkl

Additional supplementary materials:  crystallographic information; 3D view; checkCIF report

## Figures and Tables

**Table 1:** Hydrogen-bond geometry (Å, °)

*D*—H⋯*A*	*D*—H	H⋯*A*	*D*⋯*A*	*D*—H⋯*A*
O1*W*—H1⋯O3	0.95 (3)	1.82 (3)	2.764 (2)	171 (3)

**Fig. 1.**
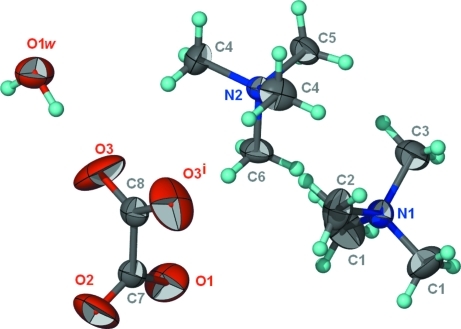
Thermal ellipsoid plot (Barbour, 2001) of 2[(CH3)4N](C2O4).H2O at the 50% probability level; hydrogen atoms are drawn as spheres of arbitrary radius.

## supplementary crystallographic information

### Experimental

Oxalic acid (0.126 g, 1 mmol) was dissolved in a water-ethanol (1:2 *v*/*v*) mixture and a 25% solution of tetramethylammonium hydroxide was added to neutralize the acid. Colorless block crystals were separated after several weeks.

### Refinement

Carbon-bound H-atoms were placed in calculated positions (C—H 0.96 Å) and were included in the refinement in the riding model approximation, with *U*(H) set to 1.5*U*(C). The water H-atom was freely refined.

### Crystal data

**Table:**

2C_4_H_12_N^+^·C_2_O_4_^2^^−^·H_2_O	*F*(000) = 560
*M**_r_* = 254.33	*D*_x_ = 1.180 Mg m^−^^3^
Orthorhombic, *P**n**m**a*	Mo *K*α radiation, λ = 0.71073 Å
Hall symbol: -P 2ac 2n	Cell parameters from 1201 reflections
*a* = 24.614 (4) Å	θ = 2.5–25.0°
*b* = 6.738 (1) Å	µ = 0.09 mm^−^^1^
*c* = 8.633 (2) Å	*T* = 293 K
*V* = 1431.8 (4) Å^3^	Block, colorless
*Z* = 4	0.50 × 0.10 × 0.10 mm

### Data collection

**Table:**

Bruker APEX2 diffractometer	1043 reflections with *I* > 2σ(*I*)
Radiation source: fine-focus sealed tube	*R*_int_ = 0.024
graphite	θ_max_ = 25.0°, θ_min_ = 2.5°
φ and ω scans	*h* = −29→11
3915 measured reflections	*k* = −8→8
1367 independent reflections	*l* = −10→8

### Refinement

**Table:**

Refinement on *F*^2^	Secondary atom site location: difference Fourier map
Least-squares matrix: full	Hydrogen site location: inferred from neighbouring sites
*R*[*F*^2^ > 2σ(*F*^2^)] = 0.053	H atoms treated by a mixture of independent and constrained refinement
*wR*(*F*^2^) = 0.164	*w* = 1/[σ^2^(*F*_o_^2^) + (0.0847*P*)^2^ + 0.5757*P*] where *P* = (*F*_o_^2^ + 2*F*_c_^2^)/3
*S* = 1.01	(Δ/σ)_max_ = 0.001
1367 reflections	Δρ_max_ = 0.25 e Å^−^^3^
99 parameters	Δρ_min_ = −0.25 e Å^−^^3^
0 restraints	Extinction correction: *SHELXL97* (Sheldrick, 2008), Fc^*^=kFc[1+0.001xFc^2^λ^3^/sin(2θ)]^-1/4^
Primary atom site location: structure-invariant direct methods	Extinction coefficient: 0.022 (5)

### Fractional atomic coordinates and isotropic or equivalent isotropic displacement parameters (Å^2^)

**Table:**

	*x*	*y*	*z*	*U*_iso_*/*U*_eq_	Occ. (<1)
O1	0.29989 (10)	0.7500	0.4672 (3)	0.0944 (10)	
O2	0.38001 (14)	0.7500	0.3510 (3)	0.1103 (12)	
O3	0.38835 (9)	0.5888 (4)	0.6773 (3)	0.1216 (10)	
O1W	0.40771 (12)	0.2500	0.8491 (3)	0.0790 (8)	
H1	0.3983 (11)	0.358 (4)	0.784 (3)	0.098 (9)*	
N1	0.04672 (8)	0.7500	0.7763 (3)	0.0442 (6)	
N2	0.27226 (8)	0.7500	1.0044 (2)	0.0372 (6)	
C1	0.02478 (11)	0.5681 (4)	0.7022 (3)	0.0864 (9)	
H1A	−0.0141	0.5665	0.7121	0.130*	
H1B	0.0397	0.4531	0.7522	0.130*	
H1C	0.0345	0.5669	0.5945	0.130*	
C2	0.10673 (11)	0.7500	0.7595 (5)	0.0679 (10)	
H2A	0.1218	0.8579	0.8181	0.102*	0.50
H2B	0.1161	0.7654	0.6522	0.102*	0.50
H2C	0.1211	0.6267	0.7974	0.102*	0.50
C3	0.03268 (14)	0.7500	0.9431 (3)	0.0652 (9)	
H3A	−0.0060	0.7379	0.9548	0.098*	0.50
H3B	0.0447	0.8719	0.9894	0.098*	0.50
H3C	0.0502	0.6402	0.9932	0.098*	0.50
C4	0.30685 (10)	0.5697 (3)	1.0196 (3)	0.0620 (7)	
H4A	0.3247	0.5708	1.1185	0.093*	
H4B	0.3336	0.5689	0.9387	0.093*	
H4C	0.2846	0.4533	1.0113	0.093*	
C5	0.22984 (12)	0.7500	1.1282 (3)	0.0522 (8)	
H5A	0.2459	0.7116	1.2249	0.078*	0.50
H5B	0.2016	0.6577	1.1014	0.078*	0.50
H5C	0.2147	0.8807	1.1377	0.078*	0.50
C6	0.24524 (12)	0.7500	0.8504 (3)	0.0548 (8)	
H6A	0.2219	0.8636	0.8423	0.082*	0.50
H6B	0.2241	0.6311	0.8391	0.082*	0.50
H6C	0.2723	0.7554	0.7704	0.082*	0.5
C7	0.35047 (12)	0.7500	0.4666 (3)	0.0486 (7)	
C8	0.37795 (10)	0.7500	0.6197 (3)	0.0479 (7)	

### Atomic displacement parameters (Å^2^)

**Table:**

	*U*^11^	*U*^22^	*U*^33^	*U*^12^	*U*^13^	*U*^23^
O1	0.0651 (17)	0.124 (3)	0.0938 (19)	0.000	−0.0318 (14)	0.000
O2	0.134 (3)	0.146 (3)	0.0503 (15)	0.000	0.0259 (16)	0.000
O3	0.1149 (17)	0.126 (2)	0.1237 (18)	0.0185 (14)	−0.0231 (13)	0.0734 (15)
O1W	0.113 (2)	0.0643 (16)	0.0591 (15)	0.000	−0.0145 (14)	0.000
N1	0.0386 (12)	0.0467 (13)	0.0473 (13)	0.000	0.0020 (9)	0.000
N2	0.0416 (11)	0.0366 (11)	0.0334 (11)	0.000	−0.0011 (9)	0.000
C1	0.0809 (17)	0.093 (2)	0.0849 (17)	−0.0298 (16)	0.0007 (14)	−0.0317 (15)
C2	0.0404 (15)	0.060 (2)	0.103 (3)	0.000	0.0112 (16)	0.000
C3	0.069 (2)	0.078 (2)	0.0486 (17)	0.000	0.0046 (15)	0.000
C4	0.0677 (13)	0.0551 (14)	0.0631 (13)	0.0215 (11)	−0.0031 (10)	0.0016 (11)
C5	0.0562 (17)	0.0583 (18)	0.0419 (15)	0.000	0.0097 (12)	0.000
C6	0.0586 (17)	0.071 (2)	0.0352 (14)	0.000	−0.0093 (12)	0.000
C7	0.0614 (18)	0.0383 (15)	0.0461 (15)	0.000	−0.0003 (13)	0.000
C8	0.0356 (14)	0.0592 (18)	0.0489 (15)	0.000	0.0053 (11)	0.000

### Geometric parameters (Å, °)

**Table:**

O1—C7	1.245 (4)	C2—H2B	0.9600
O2—C7	1.235 (4)	C2—H2C	0.9600
O3—C8	1.222 (2)	C3—H3A	0.9600
O1W—H1	0.95 (3)	C3—H3B	0.9600
N1—C3	1.481 (3)	C3—H3C	0.9600
N1—C2	1.484 (3)	C4—H4A	0.9600
N1—C1	1.484 (3)	C4—H4B	0.9600
N1—C1^i^	1.484 (3)	C4—H4C	0.9600
N2—C6	1.487 (3)	C5—H5A	0.9600
N2—C4	1.489 (2)	C5—H5B	0.9600
N2—C4^i^	1.489 (2)	C5—H5C	0.9600
N2—C5	1.494 (3)	C6—H6A	0.9600
C1—H1A	0.9600	C6—H6B	0.9600
C1—H1B	0.9600	C6—H6C	0.9600
C1—H1C	0.9600	C7—C8	1.484 (4)
C2—H2A	0.9600	C8—O3^i^	1.222 (2)
			
C3—N1—C2	109.1 (3)	N1—C3—H3C	109.5
C3—N1—C1	109.52 (16)	H3A—C3—H3C	109.5
C2—N1—C1	108.66 (17)	H3B—C3—H3C	109.5
C3—N1—C1^i^	109.52 (16)	N2—C4—H4A	109.5
C2—N1—C1^i^	108.66 (17)	N2—C4—H4B	109.5
C1—N1—C1^i^	111.3 (3)	H4A—C4—H4B	109.5
C6—N2—C4	109.54 (13)	N2—C4—H4C	109.5
C6—N2—C4^i^	109.54 (13)	H4A—C4—H4C	109.5
C4—N2—C4^i^	109.3 (2)	H4B—C4—H4C	109.5
C6—N2—C5	109.1 (2)	N2—C5—H5A	109.5
C4—N2—C5	109.68 (14)	N2—C5—H5B	109.5
C4^i^—N2—C5	109.68 (14)	H5A—C5—H5B	109.5
N1—C1—H1A	109.5	N2—C5—H5C	109.5
N1—C1—H1B	109.5	H5A—C5—H5C	109.5
H1A—C1—H1B	109.5	H5B—C5—H5C	109.5
N1—C1—H1C	109.5	N2—C6—H6A	109.5
H1A—C1—H1C	109.5	N2—C6—H6B	109.5
H1B—C1—H1C	109.5	H6A—C6—H6B	109.5
N1—C2—H2A	109.5	N2—C6—H6C	109.5
N1—C2—H2B	109.5	H6A—C6—H6C	109.5
H2A—C2—H2B	109.5	H6B—C6—H6C	109.5
N1—C2—H2C	109.5	O2—C7—O1	126.3 (3)
H2A—C2—H2C	109.5	O2—C7—C8	116.8 (3)
H2B—C2—H2C	109.5	O1—C7—C8	116.9 (3)
N1—C3—H3A	109.5	O3^i^—C8—O3	125.5 (3)
N1—C3—H3B	109.5	O3^i^—C8—C7	117.26 (17)
H3A—C3—H3B	109.5	O3—C8—C7	117.26 (17)
			
O2—C7—C8—O3^i^	89.9 (2)	O2—C7—C8—O3	−89.9 (2)
O1—C7—C8—O3^i^	−90.1 (2)	O1—C7—C8—O3	90.1 (2)

Symmetry codes: (i) *x*, −*y*+3/2, *z*.

### Hydrogen-bond geometry (Å, °)

**Table:**

*D*—H···*A*	*D*—H	H···*A*	*D*···*A*	*D*—H···*A*
O1W—H1···O3	0.95 (3)	1.82 (3)	2.764 (2)	171 (3)
